# Population structure in Atlantic cod in the eastern North Sea-Skagerrak-Kattegat: early life stage dispersal and adult migration

**DOI:** 10.1186/s13104-016-1878-9

**Published:** 2016-02-03

**Authors:** Carl André, Henrik Svedäng, Halvor Knutsen, Geir Dahle, Patrik Jonsson, Anna-Karin Ring, Mattias Sköld, Per Erik Jorde

**Affiliations:** Department of Marine Sciences-Tjärnö, University of Gothenburg, 452 96 Strömstad, Sweden; Department of Aquatic Resources, Institute of Marine Research, Swedish University of Agricultural Sciences, 453 21 Lysekil, Sweden; Institute of Marine Research, Flødevigen, 4817 His, Norway; Institute of Marine Research, PO Box, 1870, 5817 Nordnes, Bergen, Norway; Department of Biosciences, Centre for Ecological and Evolutionary Synthesis, University of Oslo, P.O. Box 1066, Blindern, 0316 Oslo, Norway; University of Agder, 4604 Kristiansand, Norway

**Keywords:** *Gadus morhua*, Larval drift, Philopatric behaviour, Population structure, Stock, Connectivity, Genetic assignment

## Abstract

**Background:**

In marine fish species, where pelagic egg and larvae drift with ocean currents, population structure has been suggested to be maintained by larval retention due to hydrographic structuring and by homing of adult fish to natal areas. Whilst natal homing of adults has been demonstrated for anadromous and coral reef fishes, there are few documented examples of philopatric migration in temperate marine fish species.

**Results:**

Here, we demonstrate temporally stable genetic differentiation among spawning populations of Atlantic cod (*Gadus morhua* L.), and present genetic and behavioural evidence for larval drift and philopatric migration in the eastern North Sea-Skagerrak-Kattegat area. We show that juvenile cod collected in the eastern Skagerrak and central Kattegat are genetically similar to cod from offshore spawning areas in the eastern North Sea. Genetic assignment of individual 2–5 year old fish indicates that cod residing at, or migrating towards, spawning areas in Kattegat and the North Sea display philopatric behaviours.

**Conclusions:**

Together these findings suggest a loop between spawning, larval drift and adult return-migrations to spawning areas and underlines that both oceanographic processes and migratory behaviour in the adult phase may be important for stock separation and integrity in marine temperate fishes such as Atlantic cod.

**Electronic supplementary material:**

The online version of this article (doi:10.1186/s13104-016-1878-9) contains supplementary material, which is available to authorized users.

## Background

Recognizing temporal and spatial structuring in marine fishes and understanding of the mechanisms responsible for population structure are of vital importance for fisheries management and species conservation [1–4]. Structuring mechanisms include fish migratory behaviour [5], in particular spawning site fidelity such as natal homing of mature adults [6], and physical forcing, where hydrodynamic stratification, currents and eddies leads to dispersal, retention or settlement of eggs and larvae [7–10]. Interactions between oceanographic and environmental features, such as bathymetry, temperature and salinity, with egg buoyancy and larval behaviour are thus considered to be of high importance for connectivity and gene flow, as they set the limits for population differentiation [11].

Because marine fish species often show ontogenetic and seasonal shifts in habitat use, the entire life cycle has to be considered when studying processes shaping population structure [12–14]. Spawning-site fidelity, i.e., adult fish returning repeatedly to spawn at the same location, irrespectively whether they were hatched at this location or not, is widely recognized in many species, e.g., Atlantic Cod, *Gadus morhua* [5]. Natal homing, where fish actively return and spawn where they were born, on the other hand, has so far been demonstrated only in a few cases, such as coral reef fishes [15–18], salmonids [19] and Atlantic bluefin tuna, *Thunnus thynnus*, [20], although strong circumstantial evidence has been presented for e.g., eels, *Anguilla* sp. [21], Atlantic herring, *Clupea harengus*, [22], pike, *Esox lucius*, [23] and Atlantic cod [24–27]. The distinction between spawning site fidelity and natal homing is fundamental because natal homing behaviour is expected to contribute to population differentiation given sufficient time, whereas spawning-site fidelity may not necessarily lead to reproductive isolation if there is extensive egg and larval drift together with opportunistic and non-philopatric recruitment of juveniles to adult aggregations [13, 28]. Demonstrating natal homing in marine fish species that disperse both passively as planktonic larvae, and by active locomotion as adults, is methodologically challenging and requires that individuals can be tracked from the egg stage to maturation and reproduction, or that reproducing individuals can be assigned to specific spawning populations using distinguishable natal tags [12, 29]. To date, natal homing has been inferred using natural tags such as elemental [6, 15, 20, 23, 30] or genetic fingerprints [17, 31].

Atlantic cod is a marine fish that exhibits population structuring on both large and small spatial scales [32, 33]. In the North Sea-Skagerrak-Kattegat area Atlantic cod comprise a mixture of co-existing resident forms completing their entire life cycle in fjords or sheltered areas [34], and oceanic populations [25, 30, 35–39]. While adult cod abundance has declined dramatically in the eastern inshore Skagerrak and Kattegat since the 1980′s, juvenile cod show no such trend [36, 40]. Genetic analyses [34, 41] in combination with oceanographic modelling [42] suggest an extensive drift of cod larvae from the North Sea into coastal Skagerrak, where they settle and possibly mix with juveniles of local coastal origin. The inflow of larvae does not, however, seem to support the diminishing coastal Skagerrak or Kattegat stocks [36, 40]. Rather, it has been hypothesized that the present low abundance of adult cod in eastern Skagerrak is due to return migrations to natal areas in the North Sea [25, 43–45].

Here, we further investigated the population structure and the mechanisms of dispersal and connectivity between cod populations in the eastern North Sea, Skagerrak and Kattegat. First, we investigated the spatial and temporal genetic structure by targeting adult fish at spawning. Second, we examined, by genetic analyses, the temporal variation in larval drift from the eastern North Sea by genetic analyses of juvenile cod in eastern Skagerrak and Kattegat collected in 2005 and 2011. Third, as both a temporally stable population structure was observed, and juveniles were found to be dispersed between areas, it was concluded that alternative structuring mechanisms to physical forcing are likely to be at play for cod in the area. For that reason, we tested whether philopatric migration behaviour, including natal homing, may act as a stock structuring process in cod by matching DNA profiles of individual migrating fish with the genetic information from cod populations on spawning grounds. Using archival tags, Svedäng et al. [25] showed that 2–5 year old cod released in eastern Skagerrak undertake directional migration towards the North Sea during the spawning season, indicating migration to potential natal spawning grounds [45]. Moreover, Svedäng et al. [25] identified groups of cod with both directional and resident migratory behaviours. By combining information from behavioural groups identified by data storage tags, with individual genetic analyses, we were able to test specifically the hypothesis that cod return to a likely place of birth at the time of spawning. This combination of methods thus elucidates the links between different life stages, and provides new insights on how population structures in marine systems are maintained and may evolve.

## Results

### Population structure

When testing for overall population divergence among the 15 adult and juvenile samples, we found a low but overall significant level of divergence (8 loci: *F*_ST_ = 0.0027; *P* = 0001: Additional file 1: Table S1). The level of divergence was fairly similar among loci (cf. Additional file 1: Table S1) and no statistical “outlying” loci were detected (Additional file 2: Figure S1), indicating absence of strong selective forces acting on these loci. Hence, they were considered as suitable for population analyses. The analysis of pairs of samples (Additional file 3: Table S2) show that the two main oceanic populations of cod, in the North Sea and Kattegat, respectively, are clearly genetically divergent (mean pairwise differentiation between the two regions, *F*_ST_ ± CI_0.95_ = 0.0036 ± 0.0013; Additional file 4: Table S3). This divergence appears temporally stable, as evidenced by samples collected in different years (Additional file 4: Table S3a), and from comparisons of year-classes (Additional file 4: Table S3b). These findings represent key requirements for statistical assignment of behavioural groups to population of origin (below).

### Juvenile cod

**Fig. 1:**
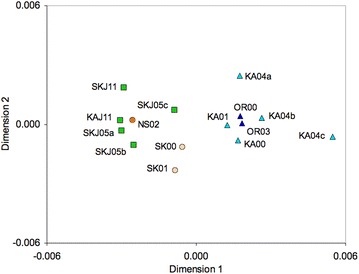
Multi-dimensional scaling plot. Based on pairwise *F*_ST_ for adult spawning populations collected in the North Sea (*orange*), western Skagerrak (*yellow*), Kattegat (*light blue*) and Öresund (*dark blue*), and juvenile 0-group cod collected in eastern Skagerrak and Kattegat (*green*). Cod samples are as in Table 2 and Fig. 3

Another important observation from these pairwise comparisons (Additional file 3: Table S2) is that juvenile 0-group cod samples collected along the Eastern Skagerrak coast in 2005 (samples SKJ05a, b, and c) and offshore in 2011 (SKJ11 and KAJ11) were all rather similar to adult eastern North Sea cod (cf. Additional file 3: Table S2), and clustered together with them (Fig. 1).

### Genetic assignment of tagged fish

**Fig. 2:**
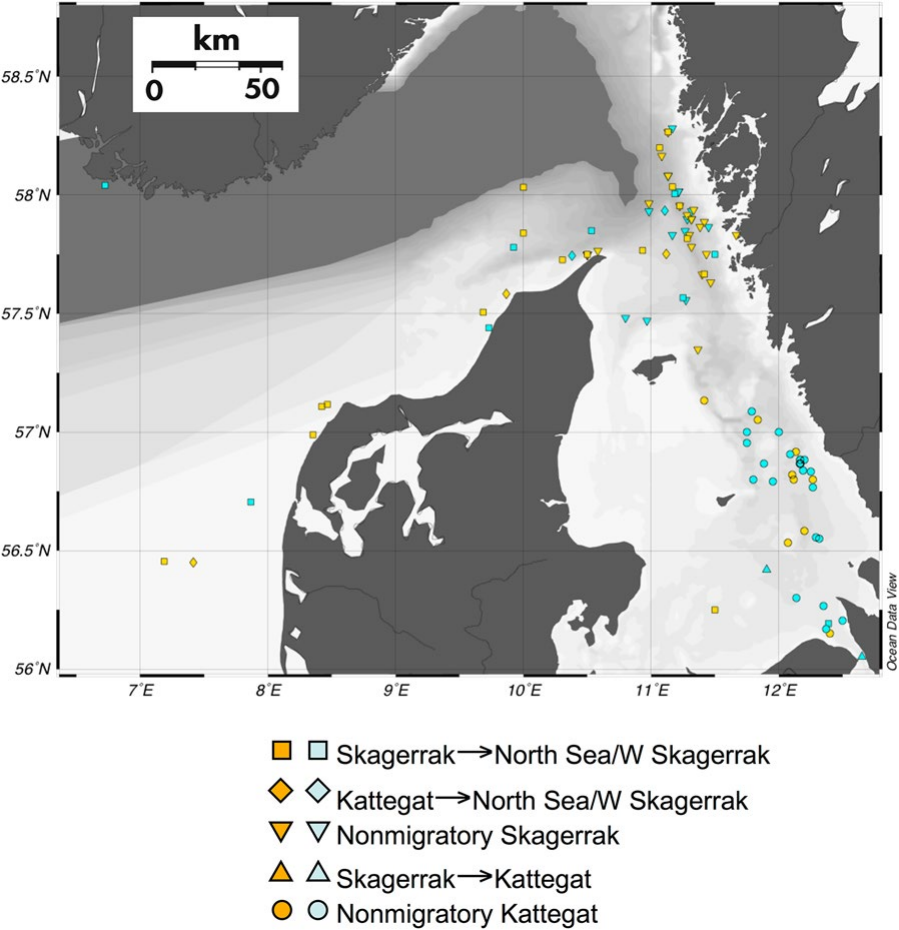
Positions of recaptured individual tagged cod. *Symbol shape* denotes migratory behaviour. Note that some fish that were geolocated west of 10°E and thus considered to have been migrating towards the North Sea were recaptured further east. *Yellow* denotes individuals genetically assigned to the pooled North Sea/W Skagerrak spawning populations and *blue* individuals assigned to the pooled Kattegat spawning populations. The map was constructed using Ocean Data View (Schlitzer R, Ocean Data View, http://www.odv.awi.de)

Assignment of adult reference individuals collected in spawning areas showed that about 80 % (83 % for the Kattegat and 79 % for the North Sea) of the fish were assigned back to their original reference population (Table 1: upper rows). The exclusion test showed that all 100 tagged individuals had a probability of 13 % or more (median 70 %) to be encountered in at least one of the two reference populations, and hence all tagged individuals were included in further analyses. The recapture positions and the genetic assignment of the tagged fish (either to the North Sea/western Skagerrak reference or to the Kattegat reference) with five different migratory behaviours are shown in Fig. 2. For the two groups of fish that migrated towards the North Sea, 60 and 63 % of the individuals were assigned to the North Sea reference population (Table 1a). All fish (3 individuals) that migrated from the Skagerrak towards the Kattegat were assigned to the Kattegat reference. A majority (67 %) of the fish that were proposed to be resident in the Kattegat were also assigned genetically to the Kattegat reference (Table 1a). Similarly, 62 % of the resident fish in the Skagerrak were assigned to the North Sea/western Skagerrak reference. Of the 100 tagged fish, 35 individuals showed directional migration in the geolocation data. An exact test showed that the direction of migration was not independent of assignment (Table 1b; *P* = 0.032), and a higher proportion than random migrated towards the population they were genetically assigned to. This finding is consistent with both philopatry (spawning site fidelity) and natal philopatry (spawning site fidelity to their “birth” site) in the migrating fish.

**Table 1:** Genetic individual assignment of tagged cod in groups with different migratory behaviours

Group	Numbers			Proportions	
	Kattegat	North Sea	Sum	Kattegat	North Sea
a					
*Reference samples:*					
Kattegat	359	76	435	0.83	0.17
North Sea/W Skagerrak	43	158	201	0.21	0.79
*Behavioural groups:*					
Skagerrak → North Sea	10	17	27	0.37	0.63
Kattegat → North Sea	2	3	5	0.40	0.60
Skagerrak → Kattegat	3	0	3	1.00	0.00
Nonmigratory Skagerrak	11	18	29	0.38	0.62
Nonmigratory Kattegat	24	12	36	0.67	0.33
b					
Assignment to	Migration towards				
	Kattegat	North Sea			
Kattegat	3	12			
North Sea/W Skagerrak	0	20			

**Fig. 3:**
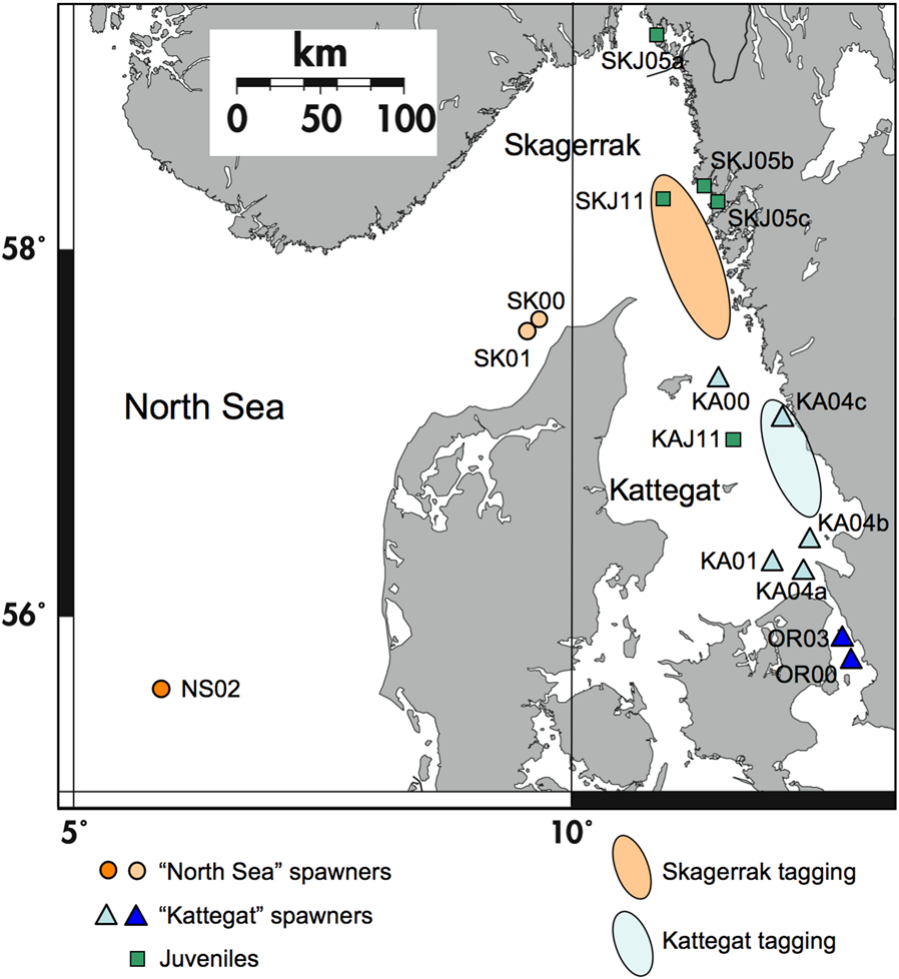
Sampling locations. Adult cod spawning samples collected in the North Sea, western Skagerrak, Kattegat and Öresund, and juvenile cod collected in the eastern Skagerrak and Kattegat. (cf. Table 2). *Orange* and *blue* *ovales* denote areas where 100 individual 2–5 y old cod were tagged and released in the eastern Skagerrak and Kattegat. See Fig. 2 for recapture locations. Map courtesy of Institute of marine research Flødevigen www.imr.no used with permission

Each of the in total 100 tagged cod were assigned to either the Kattegat reference or to the North Sea/W Skagerrak spawning reference. For definitions of groups see Table 2, Fig. 3 and text. Self-assignment of reference samples to either Kattegat or North Sea/W Skagerrak was performed by leaving the assigned individual out of the sample

Summarizing assignment for the 35 migrating individuals from part a. An exact test of independence showed that fish were more likely to migrate towards the population they were genetically assigned to (P = 0.032), indicating philopatric behaviour in the migrating fish

To assess whether the unequal sizes of the North Sea (n = 201) and Kattegat (n = 435) reference samples affected assignment power, the Kattegat reference was subsampled at n = 201 ten times (equal to the North Sea sample) and the assignment of the tagged fish was accordingly repeated. The results were similar to the original pattern, although somewhat less pronounced (compare Additional file 5: Table S4 with Table 1). Hence, there was no indication of bias towards the larger Kattegat sample.

## Discussion

The genetic assignment of juveniles in this study provides evidence of recurrent transport of Atlantic cod, most likely at the pelagic egg and larval stages, from the North Sea to coastal Skagerrak and Kattegat, in agreement with previous findings [41, 42]. Despite the obvious potential for gene flow caused by this transport, there still remains a weak but temporally stable genetic population structure of cod in the eastern North Sea-Skagerrak-Kattegat region ([34, 46–50], this study). The association between adult individual migratory behaviour and genetic assignments reported herein suggests that natal homing may act as a governing mechanism for maintaining the cod stock structure even on a relatively small spatial scale.

### Migratory behaviour

The tagged fish displayed a variety of migratory and resident behaviours, as has been shown previously in Atlantic cod [5]. The numbers of fish in the five different behavioural groups were relatively low and the results should accordingly be interpreted with caution. Nevertheless, our results show that cod with different behaviour mainly act as predicted under the hypothesis of philopatry. The majority of cod migrating towards the western Skagerrak and the North Sea were genetically assigned to the North Sea spawning population, whereas the majority of cod migrating towards the Kattegat or remaining stationary within Kattegat were assigned to the Kattegat spawning population. Cod that remain along the eastern Skagerrak during the course of this study assigned predominantly to the North Sea population, as may be hypothesised from consideration of North Sea larval drift and poor local recruitment in this area. These observations were done despite relatively low genetic differentiation between the Kattegat and the North Sea reference populations, as exemplified by the moderate self-assignment proportions for the two reference samples (cf. Table 1) and the low *F*_ST_ between them (0.0041). Such levels of genetic differentiation have however been suggested to be of clear biological relevance [34], are typical of cod also in other regional studies [26, 35, 51], and are indicative of gene flow among populations and/or a recent common historical origin. Gene flow can arise from straying of adults among spawning areas [13] or from larval drift, if some settled cod enter the local population [42]. Historically, Atlantic cod colonised the Skagerrak-Kattegat area less than 8–10,000 YBP and most populations are evolutionary young [52, 53]. Low differentiation between reference populations reduces the power of the genetic assignment tests [54], and is a likely cause for the low assignment proportions that we observed for the recaptured cod (typically 80 % self assignment in reference populations and around 65 % for each behaviour group: Table 1a and Additional file 5: Table S4). As a comparison, in a recent study of Atlantic salmon with an order of magnitude higher population divergence (*F*_ST_ ≈ 0.07), self assignment was 65 % using 14 microsatellite loci and ten source populations (n = 50) [55]. Hence, low assignment proportions of tagged fish should not necessarily be interpreted as evidence for high straying or low natal fidelity in the present study. Instead, the tagged fish are expected to show a somewhat less clear picture than the reference fish, which were specifically targeted at spawning sites at spawning time. As indicated by the exclusion tests, some of the tagged fish may have originated from populations outside the two reference groups, and this would reduce the assignment proportions. Other issues that could affect the results include the possibility that some of the eleven fish that were tagged during spring/summer had not yet started the spawning migration when recaptured in the autumn, and therefore were not classified in the correct behavioural group. Also, some groups consisted of a few individuals only (Table 1). Nevertheless, the proportions of fish in the five behaviour groups that were assigned to the expected population of origin was only slightly lower than the self assignment proportions of individual members in those populations, and in all five cases the majority of fish behaved in accordance with philopatry.

In salmonids, the evolutionary most important factor favouring natal homing has been suggested to be that such behaviour return locally adapted individuals to suitable habitats [19]. For a marine fish such as the Atlantic cod, it appears that certain areas are more suitable for spawning and that these sites are used both by migratory and resident stocks [5, 26, 56–59]. The proximate causes for the natal homing behaviour in cod are still unknown, although recent genome scan studies show that certain genomic regions are associated with migratory behaviour in cod, indicating a genetic basis for migration [39, 60]. The migratory patterns revealed in the present study seem to exclude simple environmental cues, such as prevailing directions of sea currents [61]. Experiments with salmonids have demonstrated the localization of the “natal stream” in anadromous species to be part of a learning process during the juvenile phase [62]; while small-scale localization rely on chemical cues, large scale orientation is suggested to involve geomagnetic imprinting [63, 64]. A recent experimental study on Sea turtles indicates that the magnetic environment during early development, already at the egg stage, can influence the magnetic orientation behaviour in subsequent life stages [65]. Further, the mechanisms behind natal homing has been extensively studied in tropical fish and recent modelling studies indicate that recruitment to natal reefs are expected to be relatively high and depending on the combination of sensory abilities and oceanographic features [18, 66]. For Atlantic herring, it has been suggested that migration between spawning, wintering and feeding grounds is a socially transferred behaviour, where new year classes adopt the same migratory patterns as older herring cohorts [67]. However, mixing during the juvenile stages and the subsequent migration to different spawning grounds harbouring genetically distinct populations [22, 68], suggests segregation also by natal homing in herring. For marine fishes like Atlantic herring and Atlantic cod, the stock structure may thus be imprinted already in early life stages, before juveniles of different origins intermingle in nursery areas.

### Population structure in the North Sea-Skagerrak-Kattegat

The pattern emerging from this and previous studies on cod population structure in the North Sea-Skagerrak-Kattegat region can be summarised as follows: Cod eggs and larvae are transported with currents from the North Sea to coastal Skagerrak. Juvenile cod collected in the coastal Skagerrak in 2001, 2004, 2005 and 2011 were more similar genetically to North Sea spawning aggregations than to local adult cod ([34, 41, this study Fig. 1]). Here we present new evidence that larval transport also protrude into the Kattegat (Fig. 1). The strength and direction of larval drift are apparently governed by an interaction between the size and location of the North Sea cod spawning biomass and the sea-current strength from the North Sea into the Skagerrak/Kattegat during and after spawning, and has a significant effect on the abundance of juvenile cod along the coast [40, 42]. Heath et al. [69] showed that North Sea cod consists of two genetically distinct units. While our study, and also previous genetic and modelling, have considered the Dogger unit in the central North Sea, recent work using genetic assignment tests on juvenile cod collected in 2014 in Skagerrak and northern Kattegat suggest that both Dogger Bank and the Viking Bank may be important sources of larvae for the area (J. Hemmer Hansen *unpubl. data*). Nevertheless, as cod larvae are transported into coastal fjord systems, they might be trapped by oceanographic forces, and mixed together with local coastal cod larvae [70]. On the Norwegian Skagerrak coast small local genetically distinct fjord populations persist in spite of this extensive larval drift [34, 47], indicating that foreign larvae are not incorporated into local coastal populations. The distribution pattern of juveniles and adults [36, 40, 44] and tagging studies [25, 43] suggest instead that many juveniles that grow up in coastal Skagerrak migrate back towards the North Sea when reaching maturity, at 2–3 years age; this return migration is corroborated by the genetic assignment of tagged fish presented here. In addition, we find a similar philopatric movement of cod of presumed Kattegat origin. Although conclusive evidence have been lacking, indirect evidence of natal homing have been found in several other cod populations in the eastern [24, 26, 39, 59] and western Atlantic [27, 56, 57].

## Conclusions

The distribution of adult fish in the eastern North Sea region can be regarded both as a result of spawning aggregation in certain areas and the return migrations linked to larval drift from these reproduction units. If natal homing is an important mechanisms for cod migration, as our results suggest, an area currently depleted of its local stock components can be repopulated by fish from adjacent areas only slowly, as straying adults aggregate at new locations and give rise to new populations [13, 59, 71]. The disappearance and slow recovery of local stocks of cod in the North Sea region [28, 36, 44, 72] and in the Northwest Atlantic [73, 74] is illustrative in this sense. Even if some inshore enclaves have shown signs of recovery, adjacent areas formerly equally productive, have not been repopulated [74]. Recognition of a possibly slow-changing behavioural stock structuring mechanism is hence of high importance for the assessment and management of marine fisheries [4, 75].

## Methods

### Migratory behaviour of tagged cod

Fish used for tagging were captured either in fishing pots or in trawling sets (maximum duration of 30 min). The fish were retained in tanks supplied with running seawater long enough to determine if they were in suitable condition for tagging. Typically, these were cod that could maintain buoyancy near the bottom of the tank without apparent difficulty and without external injury, such as bloodied fins or net-marks. Healthy cod were then measured to the nearest cm total length. Previous studies have suggested that, while the capture procedure may induce re-equilibration behaviour as a result of the tagging procedure [76], individuals are likely to return to normal behavioural patterns within 2 weeks of release. All tagged cod were larger than 37 cm and due to the exclusive use of the small archival tag Lotek Model LTD 2410 (http://www.lotek.com), the weight of the tag never exceeded 2.0 % of the fish body weight. Archival tags recording temperature, pressure and light intensity were deployed on 2–5 year old cod between 2003 and 2005 on the Swedish west coast. At release, records on length, weight, sex (if possible) and GPS positions were taken. All tagging was conducted under governmental licence and in adherence with national regulations on the treatment of experimental animals.

In all, we captured, tagged and released 417 individual cod, 142 in the Kattegat and 275 in the eastern Skagerrak. The archival tag was equipped with a real time clock set to UTC, a pressure sensor, external and internal temperature sensors (i.e., inside the capsulation), and a light intensity sensor. The tags were programmed to telescope the data; i.e., once the memory capacity was exhausted, some of the data were overwritten in a specified linear manner. This allows the total storage capacity of the tag to be evenly distributed over the entire mission, without sacrificing the temporal resolution of the data during the logging period. The retained data were saved in blocks of minimum 48 h. The tag also stored several parameter values on a daily basis during the deployment: e.g., estimated longitude and latitude, i.e., onboard processed estimates, sunrise and sunset in UTC, maximum external temperature, and maximum and minimum depths.

All fish were fin-clipped for DNA analyses and released at the location of capture. In total 162 individuals were recaptured between 2003 and 2006, mostly by commercial fishermen, out of which 100 individuals were selected for genetic analyses depending on two conditions: the period of time at liberty exceeded 30 days, and the migratory history of the individual could be unambiguously reconstructed from the archival tag information. Most of the fish finally selected were tagged in the autumn and had been in liberty over the spawning season in the winter, but some (11 out of 100) fish that where tagged in the spring and recaptured during the autumn had not.

Migratory trajectories of individual fish were obtained by retrospective inspection of recorded light intensity data: estimation of local noon (or local midnight) gives records on the longitude, whereas estimation of day length gives the latitude [77]. Three or more consecutive estimates departing more than one longitudinal degree from the previous location was considered as a new, valid geolocated longitude. In addition, the onboard tag algorithm estimated longitude by defining dusk and dawn at civic twilight (zenith equal to 93.44°) as characteristic changes in light intensity, and by recording the times at which they occurred. Due to data storage limitations at the time of deployment, not all days at liberty at sea could be retrospectively inspected. In order to evaluate the automatic onboard geolocation estimates an extended Kalman filter-tracking model was used as for sorting out geographic signals from the tag position time series [78]. The software package KFtrack 0.61 in the R statistical environment [79] was used for track estimation from day-logged positions. In this way the migration in east–west direction was reconstructed for the entire duration at liberty. These data were subsequently crosschecked and confirmed by the tidal location method [80], which also give estimates of the latitude. See Svedäng et al. [25] and Righton et al. [81] for more details about the tagging procedure and migration reconstruction.

The study material of 100 recaptured and genotyped (below) cod was divided into subsets of fish displaying common migratory patterns, including directional migration to the North Sea from Skagerrak or Kattegat, or to Kattegat from Skagerrak, and nonmigratory within Skagerrak or Kattegat, resulting in five behavioural categories (Table 1). Cod that were recaptured or geolocated west of 10 E° (see Fig. 3) were considered to be migrating towards the North Sea.

### Adult reference and juvenile sample collection

All recaptured fish were genotyped and statistically assigned to potential populations of origin using new and existing genetic data on cod [30, 41], collected from different spawning aggregations (Table 2). In the eastern Skagerrak-Kattegat area, the present major spawning grounds and thus the putative populations of origin for the tagged fish, are located in the North Sea/western Skagerrak [36, 41, 42] and in the Kattegat and in the Öresund [82, 83]. Whilst there are no known major spawning areas in eastern Skagerrak, small local fjord populations have been reported on the Norwegian coast in the northern Skagerrak [34, 47], and possibly also on the Swedish coast [84]. These aggregations were not, however, included in the present analysis for several reasons. The populations on the Norwegian coast are small and resident, and located mainly in the northern Skagerrak. On the Swedish coast, the aggregations are depleted and it is unclear if they represent spawning populations. Lastly, it has been shown that the power of the assignment procedure decrease with the number of putative source populations, given that no major contributing source has been left uncovered [54].

**Table 2:** Sample information and descriptive genetic statistics for cod adult spawning samples and juvenile aggregations

Sample	Location	Stage	Date	Lat	Long	N	*H*e(8)	*H*o(8)	*N*a(8)	*H*o(12)	*H*e(12)	*N*a(12)
KA00^a^	Kattegat	Adult	Jan-Feb 2000	56.90	11.90	77	0.78	0.77	15.6	0.75	0.74	14.3
OR00^a^	Öresund	Adult	Feb-Mar 2000	55.80	12.83	99	0.76	0.75	15.9	0.75	0.75	14.7
OR03	Öresund	Adult	Mar 2003	55.95	12.70	85	0.77	0.74	16.5			
KA01^a^	Kattegat	Adult	Jan-Feb 2001	56.50	12.27	58	0.77	0.75	13.6	0.75	0.73	12.9
KA04a^a^	Kattegat	Adult	Feb 2004	56.20	12.37	41	0.77	0.78	13.9	0.75	0.75	12.6
KA04b^a^	Kattegat	Adult	Feb 2004	56.30	12.32	60	0.76	0.76	14.4	0.75	0.75	13.4
KA04c^a^	Kattegat	Adult	Feb 2004	56.90	12.15	100	0.75	0.73	15.9	0.73	0.72	14.6
SK00^b^	W Skagerrak	Adult	Feb 2000	57.70	9.78	31	0.76	0.68	11.5	0.74	0.69	10.8
SK01^b^	W Skagerrak	Adult	Feb 2001	57.70	9.78	70	0.78	0.75	15.3	0.77	0.76	14.5
NS02^b^	North Sea	Adult	Mar 2002	55.57	5.85	100	0.77	0.75	16.5	0.76	0.75	15.8
SKJ05a	E Skagerrak	Juvenile	Jun 2005	59.14	10.80	92	0.77	0.77	17.1			
SKJ05b	E Skagerrak	Juvenile	Jun 2005	58.35	11.42	90	0.77	0.76	17.8			
SKJ05c	E Skagerrak	Juvenile	Jun 2005	58.28	11.54	94	0.76	0.74	16.6			
SKJ11	Skagerrak	Juvenile	Aug 2011	58.25	10.49	166	0.76	0.71	18.3			
KAJ11	Kattegat	Juvenile	Aug 2011	56.63	11.59	167	0.76	0.74	18.9			

Adult samples and juvenile 2011 samples were collected by trawling, and juvenile 2005 samples by beach seine

*H*_e_ average expected heterozygosity, *H*_o_ average observed heterozygosity, *N*_a_ average number of alleles for (8) and (12) microsatellite loci

^a^Pooled reference sample “Kattegat”

^b^Pooled reference sample “North Sea/W Skagerrak”

A total of nine samples of adult Atlantic cod representing the two reference groups were collected during 2000–2004 (global n = 636). Group 1, constitutes of spawning aggregations in the eastern North Sea, including the “Dogger unit” [69] and the western Skagerrak, and group 2 include the Kattegat and Öresund (Fig. 3, 1, Table 2). An additional sample of adult Öresund cod, collected in 2003, was also genotyped for comparison purposes, but was not included in the reference set (Table 2). The fish were collected during the spawning period from January to March by trawling, and care was taken to choose mature fish that were at or close to spawning. Muscle tissue or fin clips for DNA analysis were stored in ethanol.

Juvenile 0-group cod (about 6–12 cm in length) were sampled in June 2005 at three locations in the eastern coastal Skagerrak using a beach seine, and two locations in August 2011 in the Kattegat and Skagerrak by trawling during the IBTS expedition (http://www.ices.dk; Fig. 3, Table 2). All cod samples used were collected in compliance with EU Directive 2010/63/EU, and the national legislations in Sweden (Swedish Board of Agriculture (http://www.jordbruksverket.se,) permit number no. 126-2015 to Swedish University of Agricultural Sciences, Department of Aquatic Resources) and Norway (the Institute of Marine Research have permission to sample cod by the Directorate of Fisheries, Bergen, Norway). No endangered species were used in the present study.

### Genetic analyses

DNA was extracted from fin clips or muscle tissue using the dneasy animal tissue kit (Qiagen Inc.). All fish were genotyped for eight microsatellite DNA loci following published protocols with minor modifications: Gmo2 and Gmo132 [85]; Gmo3, Gmo8, Gmo19, Gmo34, Gmo35 [86]; and Tch5 [87]. To increase resolution, the tagged and reference fish were genotyped for additional four loci (i.e., in total twelve): Gmo36, and Gmo37 [86] and Tch13 and Tch22 [87]. The microsatellite DNA fragments were separated on ALF express II (Amersham Pharmacia Biotech), CEQ 8000 Genetic Analysis System (Beckman Coulter) and ABI 3730 Genetic Analyser (Applied Biosystems) automatic sequencers. Repeating two “control” individuals spanning the anticipated allelic ranges on all runs, in addition to internal and external size ladders, ensured scoring consistency among runs and platforms. Two persons scored genotypes independently and any inconsistent scorings were noted and the fish was screened again. We assessed scoring quality of the final data by searching for allele-specific departures from Hardy–Weinberg proportions at each locus, as could arise from allele dropout or null-alleles. This was done by considering one allele at a time, pooling all other alleles at the locus, and calculating *F*_IS_ for each allele separately based on genotypes thus pooled. We used the Chi square test X^2^ = n*F_IS_ [88] with df = 1, where *n* is the sample size, to assess deviations from HW proportion for that allele. However, no systematic departures were found (data not shown).

### Statistical analyses of genetic data

Heterozygosity in adult and juvenile samples was calculated using FSTAT [89] (Table 2). Population differentiation among samples was estimated using the *F*_ST_ estimator *θ* [90], and statistical significance assessed using the software genepop 4.2. [91]. The software lositan [92] was used to test for outliers among the eight loci, possibly affected by selection. The pattern of genetic differentiation was explored among the adult and juvenile samples with a Multi Dimensional Scaling plot, based on pair-wise *F*_ST_ (Additional file 3: Table S2) using cmndscale (r-project.org). The MDS plot (Fig. 1) further indicated that the nine reference samples could be pooled into two regional groups: “North Sea/W Skagerrak” (on the left side in Fig. 1) and “Kattegat” (right side); see also table of pair-wise *F*_ST_ (Additional file 4: Table S3a). The pooling of reference samples had the benefit of both reducing the number of reference populations and increasing the reference sample sizes without losing significant geographic information. We further assessed the temporal stability of the genetic differentiation between the two regional groups by dividing all fish into year classes based on otolith ageing, and testing for genetic heterogeneity (Additional file 4: Table S3b). This latter test did not indicate any temporal instability in either the North Sea/W Skagerrak or the Kattegat groups and verified the genetic integrity of the two regions. All reference samples were thus pooled into two regions North Sea/W Skagerrak and Kattegat in subsequent statistical analyses; the *F*_ST_ between the pooled North Sea/W Skagerrak (n = 201) and the pooled Kattegat reference (n = 435) was 0.0041 (*P* < 0.0001).

Genetic assignment of recaptured tagged individuals to the two pooled reference populations, as well as “self assignment” of reference individuals, was performed with the software geneclass2 [93]. The possibility that the tagged fish originated from unknown spawning populations, not covered in the reference data, was evaluated using the exclusion method in geneclass2, where the likelihood of individual fish belonging to a given reference population was compared with the distribution of likelihoods of 1000 genotypes simulated from each reference population with a Monte Carlo algorithm [94].

The most likely origin of fish in the five behavioural groups was determined by calculating the proportion of fish that were assigned to each of the two regional reference pools. For the 35 fish that showed directional migration we tested if migration was independent from genetic assignment using an unconditional exact test of independence (Boschloo’s test in the exact R-package, [95]).

### Availability of supporting data

The microsatellite data set is available in the DRYAD repository, http://datadryad.org, doi:10.5061/dryad.m3913.

## Additional files

10.1186/s13104-016-1878-9 Overall genetic differentiation (*F*_ST_) among cod samples for eight microsatellite loci.
10.1186/s13104-016-1878-9 Comparison of population differentiation (*F*_ST_) and heterozygosity (*H*_E_) among 15 samples of cod in eight microsatellite loci to identify outliers and potential candidates for selection using LOSITAN. All loci are candidates to be selectively neutral.
10.1186/s13104-016-1878-9 Pairwise genetic differentiation (*F*_ST_) among adult and juvenile cod samples.
10.1186/s13104-016-1878-9 Pair-wise genetic differentiation between cod reference samples.
10.1186/s13104-016-1878-9 Assessment of sample size in reference samples for genetic assignment of migratory fish.
