# Supplementary material for: Population structure in Atlantic cod in the eastern North Sea-Skagerrak-Kattegat: early life stage dispersal and adult migration
Source: BMC Res Notes. 2016 Feb 3;9:63. doi: 10.1186/s13104-016-1878-9 (PMC4739106; doi:10.1186/s13104-016-1878-9)
Supplement: Supplementary file 1 — 10.1186/s13104-016-1878-9 Overall genetic differentiation (F ST) among cod samples for eight microsatellite loci. [file 13104_2016_1878_MOESM1_ESM.doc]

Additional Table 1. Overall differentiation (*F*_ST_) for eight microsatellite loci for 10 adult and 5 juvenile cod samples, in total 15 samples listed in Table 1.

Locus *F*_ST_ P-value

Gmo2 0.0020 **0.0192**

Gmo3 0.0068 **0.0004**

Gmo8 0.0081 **0.0141**

Gmo19 0.0019 **0.0000**

Gmo34 0.0008 0.1811

Gmo35 0.0004 **0.0222**

Gmo132 0.0042 **0.0000**

Tch5 -0.0001 0.3778

**All: 0.0027**  **0.0001**
