# Supplementary material for: Population structure in Atlantic cod in the eastern North Sea-Skagerrak-Kattegat: early life stage dispersal and adult migration
Source: BMC Res Notes. 2016 Feb 3;9:63. doi: 10.1186/s13104-016-1878-9 (PMC4739106; doi:10.1186/s13104-016-1878-9)
Supplement: Supplementary file 2 — 10.1186/s13104-016-1878-9 Comparison of population differentiation (F ST) and heterozygosity (H E) among 15 samples of cod in eight microsatellite loci to identify outliers and potential candidates for selection using LOSITAN. All loci are candidates to be selectively neutral. [file 13104_2016_1878_MOESM2_ESM.doc]

Additional Figure 1. Comparison of population differentiation (*F*_ST_) and heterozygosity (*H*_E_) among 15 samples of cod in eight microsatellite loci to identify outliers and potential candidates for selection using LOSITAN. All loci are candidates to be selectively neutral.
