# Supplementary material for: Population structure in Atlantic cod in the eastern North Sea-Skagerrak-Kattegat: early life stage dispersal and adult migration
Source: BMC Res Notes. 2016 Feb 3;9:63. doi: 10.1186/s13104-016-1878-9 (PMC4739106; doi:10.1186/s13104-016-1878-9)
Supplement: Supplementary file 3 — 10.1186/s13104-016-1878-9 Pairwise genetic differentiation (F ST) among adult and juvenile cod samples. [file 13104_2016_1878_MOESM3_ESM.doc]

Additional Table 2. Genetic differention among adult and juvenile cod samples. Population pairwise *F*_ST_ based on 8 microsatellite loci below diagonal. *P*-values above diagonal. Bold denotes *P*<0.05.

| *F*_ST_/*P*-val | KA00 | KA01 | KA04a | KA04b | KA04c | OR00 | OR03 | NS02 | SK00 | SK01 | SKJ05c | SKJ05a | SKJ05b | KAJ11 | SKJ11 |
| --- | --- | --- | --- | --- | --- | --- | --- | --- | --- | --- | --- | --- | --- | --- | --- |
| KA00 | - | 0.2892 | 0.1315 | 0.5038 | 0.1565 | 0.2318 | 0.1122 | 0.1459 | 0.0703 | 0.1205 | **0.0003** | 0.1087 | **0.0064** | **0.0011** | **0.0000** |
| KA01 | -0.0015 | - | 0.3705 | 0.7593 | 0.5379 | 0.7806 | 0.3533 | **0.0282** | **0.0067** | 0.7473 | **0.0286** | 0.0936 | 0.0981 | **0.0275** | **0.0028** |
| KA04a | 0.0012 | 0.0005 | - | 0.4804 | 0.4693 | 0.1325 | 0.5098 | 0.0975 | **0.0343** | **0.0336** | 0.3778 | 0.0538 | 0.0591 | **0.0079** | **0.0049** |
| KA04b | -0.0006 | -0.0039 | -0.0004 | - | 0.5398 | 0.2380 | 0.2973 | **0.0079** | **0.0050** | 0.1274 | **0.0306** | **0.0072** | **0.0022** | **0.0020** | **0.0000** |
| KA04c | 0.0005 | -0.0002 | 0.0004 | -0.0008 | - | 0.1894 | 0.8248 | **0.0000** | **0.0064** | **0.0078** | **0.0004** | **0.0000** | **0.0000** | **0.0000** | **0.0000** |
| OR00 | 0.0002 | -0.0014 | 0.0002 | -0.0014 | -0.0002 | - | 0.0566 | **0.0002** | **0.0216** | **0.0291** | **0.0016** | **0.0000** | **0.0022** | **0.0001** | **0.0000** |
| OR03 | -0.0001 | -0.0006 | 0.0000 | 0.0001 | -0.0004 | -0.0005 | - | **0.0000** | **0.0213** | **0.0157** | **0.0179** | **0.0005** | **0.0070** | **0.0000** | **0.0000** |
| NS02 | 0.0032 | 0.0040 | 0.0034 | 0.0053 | 0.0078 | 0.0033 | 0.0044 | - | 0.6462 | 0.4310 | 0.7777 | 0.9064 | 0.1903 | 0.2331 | **0.0055** |
| SK00 | 0.0002 | 0.0033 | 0.0042 | 0.0040 | 0.0046 | 0.0019 | 0.0018 | -0.0019 | - | **0.0322** | 0.2042 | 0.7132 | 0.1245 | 0.1039 | 0.0527 |
| SK01 | 0.0014 | -0.0009 | 0.0057 | 0.0028 | 0.0052 | 0.0029 | 0.0033 | 0.0007 | 0.0013 | - | 0.1355 | 0.2578 | 0.3023 | **0.0241** | **0.0000** |
| SKJ05c | 0.0050 | 0.0031 | 0.0023 | 0.0029 | 0.0045 | 0.0021 | 0.0032 | -0.0006 | 0.0025 | 0.0018 | - | 0.1410 | 0.3031 | 0.0978 | **0.0230** |
| SKJ05a | 0.0028 | 0.0043 | 0.0047 | 0.0060 | 0.0082 | 0.0050 | 0.0046 | -0.0009 | -0.0014 | 0.0023 | 0.0015 | - | **0.0069** | **0.0194** | **0.0102** |
| SKJ05b | 0.0053 | 0.0042 | 0.0046 | 0.0060 | 0.0073 | 0.0056 | 0.0042 | 0.0009 | 0.0023 | 0.0013 | 0.0009 | 0.0022 | - | **0.0000** | **0.0000** |
| KAJ11 | 0.0050 | 0.0041 | 0.0047 | 0.0057 | 0.0082 | 0.0037 | 0.0048 | -0.0005 | 0.0028 | 0.0015 | -0.0001 | 0.0010 | 0.0017 | - | 0.1134 |
| SKJ11 | 0.0045 | 0.0033 | 0.0032 | 0.0053 | 0.0088 | 0.0045 | 0.0043 | 0.0007 | 0.0023 | 0.0036 | 0.0012 | 0.0007 | 0.0039 | -0.0003 | - |
