# Supplementary material for: Population structure in Atlantic cod in the eastern North Sea-Skagerrak-Kattegat: early life stage dispersal and adult migration
Source: BMC Res Notes. 2016 Feb 3;9:63. doi: 10.1186/s13104-016-1878-9 (PMC4739106; doi:10.1186/s13104-016-1878-9)
Supplement: Supplementary file 4 — 10.1186/s13104-016-1878-9 Pair-wise genetic differentiation between cod reference samples. [file 13104_2016_1878_MOESM4_ESM.doc]

Additional Table 3. Pair-wise genetic differentiation between cod reference populations from the Kattegat, Öresund, western Skagerrak and the North Sea based on 12 microsatellite loci. *F*_ST_ below diagonal and corresponding *P*-values above. **P*<0.05, ***P*<0.01, ****P*<0.001

Boxes indicate sample pairs within and between regions (Kattegat vs North sea), respectively.

a. analyses based on samples; mean pairwise differentiation between the two regions *F*_ST_ ± CI_0.95_ = 0.0036 ± 0.0013

b. analyses based on year classes (cohorts) with n>20; mean pairwise differentiation between the two regions *F*_ST_ ± CI_0.95_ = 0.0030 ± 0.0011

a.

| *F_ST_* \ *P* | KA00 | OR00 | KA01 | KA04a | KA04b | KA04c | SK00 | SK01 | NS02 |
| --- | --- | --- | --- | --- | --- | --- | --- | --- | --- |
| KA00 | - | 0.374 | 0.692 | 0.151 | 0.717 | 0.217 | 0.413 | 0.168 | 0.043* |
| OR00 | -0.0003 | - | 0.811 | 0.216 | 0.402 | 0.333 | 0.182 | 0.039* | 0.000*** |
| KA01 | -0.0021 | -0.0011 | - | 0.514 | 0.932 | 0.490 | 0.088 | 0.721 | 0.115 |
| KA04a | 0.0013 | -0.0002 | 0.0001 | - | 0.344 | 0.472 | 0.283 | 0.036* | 0.307 |
| KA04b | -0.0006 | -0.0012 | -0.0030 | -0.0004 | - | 0.646 | 0.075 | 0.077 | 0.006** |
| KA04c | 0.0004 | -0.0002 | -0.0001 | 0.0000 | -0.0010 | - | 0.029* | 0.000*** | 0.000*** |
| SK00 | -0.0008 | 0.0000 | 0.0020 | 0.0005 | 0.0020 | 0.0031 | - | 0.334 | 0.997 |
| SK01 | 0.0021 | 0.0029 | 0.0010 | 0.0044 | 0.0039 | 0.0073 | -0.0011 | - | 0.667 |
| NS02 | 0.0029 | 0.0027 | 0.0032 | 0.0019 | 0.0044 | 0.0074 | -0.0037 | -0.0002 | - |

b.

| *F_ST_* \ *P* | KA 94 | KA 96 | KA 97 | KA 98 | KA 01 | KA 02 | NS 96 | NS 98 | NS 99 |
| --- | --- | --- | --- | --- | --- | --- | --- | --- | --- |
| KA 94 (n = 22) | - | 0.697 | 0.651 | 0.753 | 0.378 | 0.573 | 0.349 | 0.032* | 0.357 |
| KA 96 (n = 57) | -0.0013 | - | 0.317 | 0.492 | 0.066 | 0.299 | 0.250 | 0.032* | 0.004** |
| KA 97 (n = 90) | 0.0008 | 0.0007 | - | 0.924 | 0.098 | 0.131 | 0.139 | 0.035* | 0.005** |
| KA 98 (n = 28) | -0.0029 | -0.0014 | -0.0005 | - | 0.764 | 0.567 | 0.212 | 0.166 | 0.411 |
| KA 01 (n = 154) | 0.0000 | 0.0011 | 0.0011 | -0.0015 | - | 0.097 | 0.001** | 0.000*** | 0.000*** |
| KA 02 (n = 29) | -0.0018 | -0.0002 | 0.0047 | -0.0022 | 0.0032 | - | 0.065 | 0.001** | 0.021* |
| NS 96 (n = 24) | 0.0002 | -0.0006 | 0.0031 | 0.0010 | 0.0045 | 0.0021 | - | 0.749 | 0.969 |
| NS 98 (n = 66) | 0.0071 | 0.0024 | 0.0047 | 0.0042 | 0.0085 | 0.0080 | -0.0035 | - | 0.425 |
| NS 99 (n = 24) | 0.0015 | 0.0025 | 0.0042 | 0.0015 | 0.0055 | 0.0050 | -0.0039 | 0.0005 | - |
