# Supplementary material for: Population structure in Atlantic cod in the eastern North Sea-Skagerrak-Kattegat: early life stage dispersal and adult migration
Source: BMC Res Notes. 2016 Feb 3;9:63. doi: 10.1186/s13104-016-1878-9 (PMC4739106; doi:10.1186/s13104-016-1878-9)
Supplement: Supplementary file 5 — 10.1186/s13104-016-1878-9 Assessment of sample size in reference samples for genetic assignment of migratory fish. [file 13104_2016_1878_MOESM5_ESM.doc]

Additional Table 4. To investigate whether the unequal sizes of the baseline samples introduce bias, we subsampled (n=201) the Kattegat reference sample ten times and repeated the assignment procedure as in table 2.

|  |  | Numbers (mean) |  |  | Proportions (mean) |  |
| --- | --- | --- | --- | --- | --- | --- |
| Group | Kattegat | North Sea | Sum | Kattegat | North Sea | Standard error |
| *Reference samples:* |  |  |  |  |  |  |
| Kattegat self - assign | 132.3 | 68.7 | 201 | 0.66 | 0.34 | 0.006 |
| North Sea self - assign | 75.1 | 125.9 | 201 | 0.37 | 0.63 | 0.007 |
| *Behavioural groups:* |  |  |  |  |  |  |
| Skagerrak→North Sea | 12.1 | 14.9 | 27 | 0.45 | 0.55 | 0.022 |
| Kattegat→North Sea | 2.2 | 2.8 | 5 | 0.44 | 0.56 | 0.058 |
| Nonmigratory Skagerrak | 11.4 | 17.6 | 29 | 0.39 | 0.61 | 0.015 |
| Skagerrak→Kattegat | 2.5 | 0.5 | 3 | 0.83 | 0.17 | 0.056 |
| Nonmigratory Kattegat | 22.6 | 13.4 | 36 | 0.63 | 0.37 | 0.022 |
